# Two cases of severe vitamin D_3_ intoxication treated with therapeutic plasma exchange and high cut-off hemodialysis

**DOI:** 10.1007/s40620-022-01543-2

**Published:** 2022-12-22

**Authors:** David J. Heister, Bernhard N. Bohnert, Nils Heyne, Andreas L. Birkenfeld, Ferruh Artunc

**Affiliations:** 1 Division of Endocrinology, Diabetology and Nephrology, Department of Internal Medicine IV, University Hospital Tübingen, Otfried-Müller-Str.10, 72076 Tübingen, Germany; 2 Institute of Diabetes Research and Metabolic Diseases (IDM) of the Helmholtz Center Munich at the University of Tübingen, Otfried-Müller-Strasse 10, 72076 Tübingen, Germany; 3 German Center for Diabetes Research (DZD), Otfried-Müller-Strasse 10, 72076 Tübingen, Germany

**Keywords:** Hypercalcemia, Vitamin D, Plasma exchange, Renal dialysis, Acute kidney injury

## Abstract

We report on a 53-year-old female patient and a 33-year-old male patient presenting with life-threatening hypercalcemic crisis caused by self-induced vitamin-D intoxication. Both patients took high doses of vitamin D_3_ supplements, cumulatively up to 2,500,000–10,000,000 I.U. over several months. Accordingly, serum 25-OH-vitamin D concentrations were increased to 663 and 1289 nmol/L (reference 50–175 nmol/L), respectively. As forced diuresis and bisphosphonates failed to correct recurrent hypercalcemia, we hypothesized that add-on extracorporeal treatments might help overcome the refractory situation. Considering the binding of vitamin D_3_ metabolites to vitamin D-binding protein (VDBP, 59 kDa), we started extracorporeal treatments involving total plasma exchange with replacement by human albumin and by fresh frozen plasma, online hemodiafiltration and high cut-off hemodialysis. We found that in the former case, total plasma exchange with albumin and fresh frozen plasma and high cut-off hemodialysis lowered both 25-OH-vitamin D_3_ and 1,25-OH-vitamin D_3,_ whereas in the latter case total plasma exchange with albumin was found to more effectively remove vitamin D metabolites compared to high cut-off hemodialysis. In contrast, the amount of total plasma calcium removed by high cut-off hemodialysis was higher compared to total plasma exchange with albumin. During follow up, patients 1 and 2 achieved almost normal total plasma calcium and vitamin D concentrations after 355 and 109 days, respectively. These two cases suggest that extracorporeal treatments with high cut-off hemodialysis and total plasma exchange with albumin may be considered as add-on treatment in refractory cases of vitamin D_3_-induced hypercalcemia to lower plasma 25-OH-vitamin D_3_ concentrations.

## Introduction

In humans, plasma calcium concentration is maintained within a very narrow range. Intestinal calcium absorption is the limiting step of calcium balance and is governed by active 1,25-OH-vitamin D_3_, which is produced by the proximal tubule after 1α-hydroxylation of 25-OH-vitamin D_3_. Typically, the plasma concentration of 25-OH-vitamin D_3_ exceeds that of 1,25-OH-vitamin D_3_ by a factor of 1000 so that 25-OH-vitamin D_3_ can be considered as a large reservoir. In plasma, 25-OH-vitamin D_3_ and 1,25-OH-vitamin D_3_ circulate bound to the vitamin D-binding-protein (VDBP), which has a molecular weight of 52–59 kDa [1].

Vitamin D can be supplemented by oral intake of cholecalciferol or vitamin D_3_, which is converted to 25-OH-vitamin D_3_ by the liver. Vitamin D intoxication and resulting hypercalcemia are rare in clinical practice [2]. Due to its high lipophilicity, 25-OH-vitamin D_3_ stores are eliminated slowly and symptom control may take several months [3]. In theory, extracorporeal treatment procedures can be considered for severe cases of vitamin D_3_ intoxication [4, 5]. However, due to its high lipophilicity and protein binding, removal of vitamin D_3_ metabolites using conventional hemodialysis is not feasible. In neurologic patients, therapeutic/total plasma exchange (TPE) has been shown to remove vitamin D_3_ metabolites [4]. Given the elimination of proteins up to 60 kDa, high cut-off hemodialysis might effectively remove vitamin D_3_ metabolites bound to VDBP from the bloodstream [6]. To our knowledge, there are no reports of severe vitamin D_3_ intoxication treated by extracorporeal removal of vitamin D_3_ metabolites. Here, we suggest that extracorporeal treatments may be considered as add-on treatment in refractory cases of vitamin D_3_-induced hypercalcemia.

## Methods

The 25-OH vitamin D concentrations in blood plasma, eluate and dialysate were measured by mass spectrometry (LC–MS/MS) using the Cascadion™ SM Clinical Analyzer (Thermo Fisher Scientific). For the determination of dialysate 25-OH vitamin D concentrations, samples were concentrated 40-fold via ultrafiltration using an Amicon^®^ Ultra-4 10 K centrifugal filter device (Merck, Germany). 1,25-OH-vitamin D3 concentration was measured using chemiluminescence immunoassay (IDS-ISYS, Immunodiagnostic System). Plasma creatinine was determined enzymatically and calcium concentration was determined using photometric endpoint determination (o-cresolphthalein complex method) on ADVIA Chemistry XPT system (Siemens Healthineers).

### Case 1

#### Patient information

A 53-year-old female was initially admitted to the hospital for treatment of relapsing progressive multiple sclerosis diagnosed 15 years earlier. She presented to our hospital with aggressive behavior, slow thinking, impaired cognition, and fatigue. In addition, she showed limited ability to walk and pain in the legs and left shoulder with radiation and weakness in the left arm. She had taken around 30,000 I.U./days cholecalciferol (vitamin D_3_) in the previous 16 weeks (total approximately 2,500,000 I.U.) in an attempt to positively impact the course of her multiple sclerosis and to compensate for vitamin D deficits, as widely communicated in the media.

#### Clinical findings

Physical examination showed normal and symmetrical reflexes, hyperactive biceps reflexes with an expanded reflex zone on both sides. There were no other previously unknown clinical findings attributable to hypercalcemia.

#### Diagnostic assessment

**Fig. 1:**
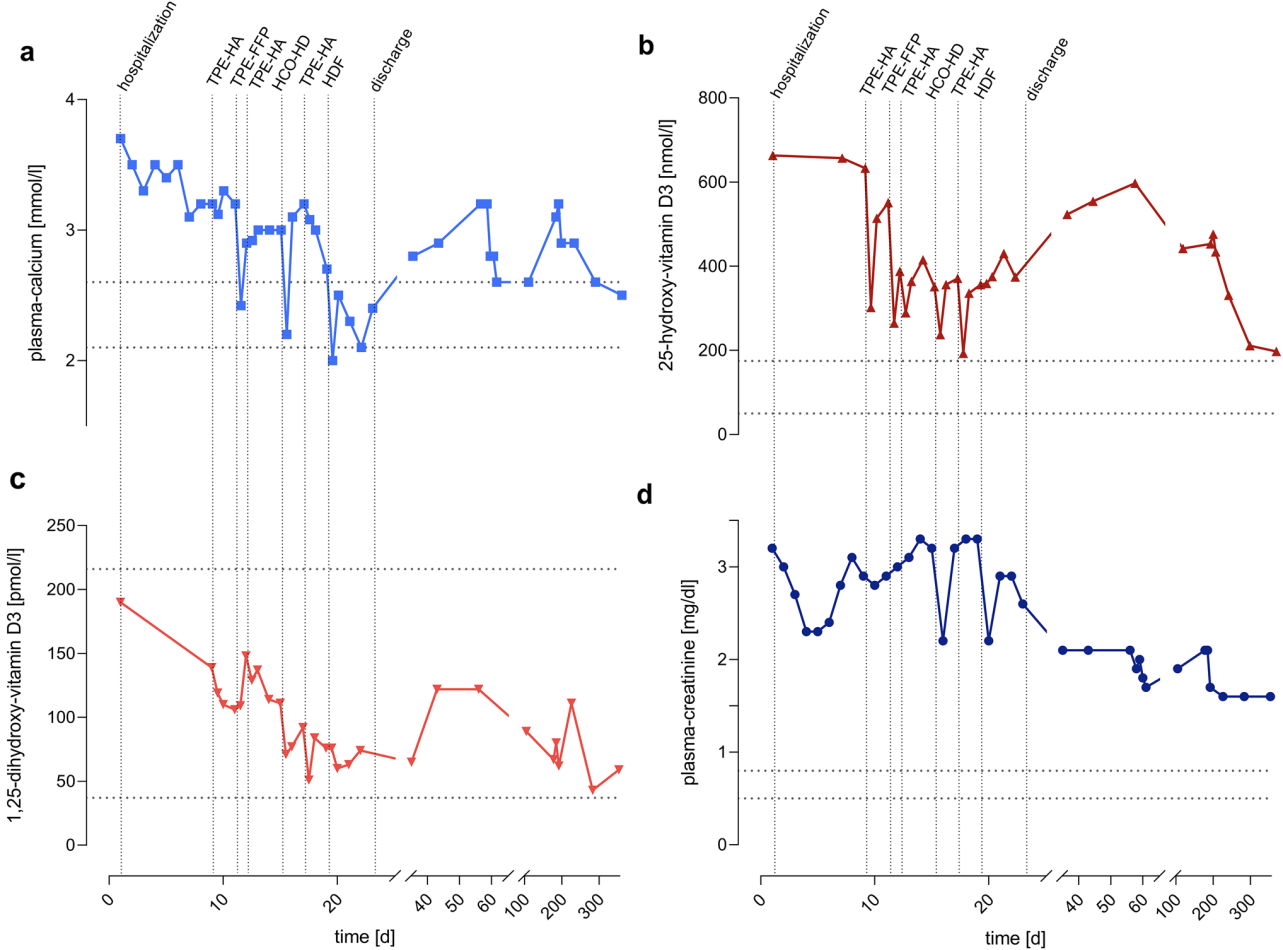
Time course of plasma concentrations of calcium (**a**), 25-OH-vitamin D_3_ (**b**), 1,25-OH-vitamin D_3_ (**c**) and creatinine (**d**) of patient 1. Hospital admission, discharge and extracorporeal treatments are marked with vertical dotted lines. Normal ranges are marked with horizontal dotted lines. *HDF* hemodiafiltration, *HCO–HD* high cut-off hemodialysis, *TPE-FFP* total plasma exchange with replacement by fresh frozen plasma, *TPE-HA* total plasma exchange with replacement by human albumin

Severe hypercalcemia was present with total and ionized plasma calcium concentrations of 3.7 mmol/L (reference 2.1–2.6 mmol/L) and 2.96 mmol/L (reference 1.14–1.29 mmol/L, Fig. 1A), respectively. The plasma concentration of 25-OH-vitamin D_3_ was highly elevated to 663 nmol/L (reference 50–175 nmol/L, Fig. 1B), while the plasma concentration of 1,25-OH-vitamin D_3_ was 190 pmol/L (reference 37–216 pmol/L, Fig. 1C). In addition, acute kidney injury with a plasma creatinine concentration of 299 µmol/L or 3.4 mg/dL (reference < 79 µmol/L or < 0.9 mg/dL, Fig. 1D) was present. No evidence of a pre-renal or post-renal condition, malignancy or primary hyperparathyroidism was present.

#### Therapeutic intervention

Upon hospital admission, the patient was treated with forced diuresis consisting of intravenous fluid replacement (2.5–5.6 L/days), torasemide (10–30 mg/days) and spironolactone (50 mg/days), which resulted in a lowering of the plasma calcium concentration to 3.1 mmol/L. As elevated concentrations of vitamin D_3_ metabolites persisted and maintained the hypercalcemia, we decided to perform therapeutic plasma exchange with 5% human albumin (TPE-HA) as replacement fluid to remove circulating vitamin D_3_ metabolites. After inserting a central venous dialysis catheter and exchanging approximately one plasma volume (2.5 L), plasma 25-OH-vitamin D_3_ concentration dropped by 52% (to 302 nmol/L, Fig. 1B) and that of 1,25-OH-vitamin D3 by 14% (to 119 pmol/L, Fig. 1C). However, 25-OH-vitamin D_3_ rebounded to 514 nmol/L. Due to low fibrinogen concentrations, we performed another total plasma exchange with 10 units (approximately 3.11 L) of fresh frozen plasma (TPE-FFP) as replacement, which lowered 25-OH-vitamin D3 by 52%, but not that of 1,25-OH-vitamin D_3_ (Fig. 1C). Repeating therapeutic plasma exchange with human albumin resulted in the reduction of both vitamin D metabolites. In the meantime, 2 mg of ibandronic acid was administered intravenously. To treat rebounding concentrations of 25-OH-vitamin D_3_ and calcium, we decided to perform HD with a high cut-off dialyzer (Table 1). Using high cut-off hemodialysis, 25-OH-vitamin D_3_ and 1,25-OH-vitamin D_3_ levels fell by 31 and 36%, respectively (Fig. 1B/C). To lower the plasma calcium concentration, we decided to perform online hemodiafiltration (HDF) with a high flux dialyzer (Table 1). As expected, no changes in 25-OH-vitamin D_3_ and 1,25-OH-vitamin D_3_ concentrations were achieved. Plasma calcium concentration finally normalized (Fig. 1A), allowing the patient to be discharged after 30 days of hospitalization. However, 25-OH-vitamin D_3_ concentration remained elevated at 374 nmol/L.

**Table 1:** Technical details of the extracorporeal treatment procedures in both patients

Procedure	Hemodiafiltration (HDF)	High cut-off hemodialysis (HCO-HD)	Total plasma exchange with fresh frozen plasma (TPE-FFP)	Total plasma exchange with 5% human albumin (TPE-HA)
Number of treatments (*n*) per patient (Pt)	1 (patient 1) 0 (patient 2)	1 (patient 1) 3 (patient 2)	1 (patient 1) 0 (patient 2)	3 (patient 1) 4 (patient 2)
Machine	Fresenius Medical Care 5008	Fresenius Medical Care 5008	Spectra Optia^*®*^* Apheresis System (Terumo BCT)*	Spectra Optia^*®*^* Apheresis System (Terumo BCT)* *or* *Octo Nova*^*®*^* (Diamed Medizintechnik)*
Filter/dialyzer	CorDiax Fx60, *A* = 1.4 m^2^ *(Fresenius Medical Care)*	Gambro Theralite 2100, *A* = 2.1 m^2^ *(Baxter International)*	Centrifugation	Centrifugation or Plasmaflow P-05 W(L) (*Asahi Kasei Medical Co., Ltd.*)
Permeability	Plasma proteins up to 20–25 kDa [8]	Plasma proteins up to 45–60 kDa [9, 10]	All plasma proteins	All plasma proteins
Anticoagulation	Heparin	Heparin	Citrate Dextrose Solution Ph Eur (ACD) Solution A *(Terumo BCT)*	Citrate Dextrose Solution Ph Eur (ACD) Solution A *(Terumo BCT)*
Exchange volume	–	–	10 FFP ≈ 3.1 L	2.5 L (patient 1) 2.5–3.5 L (patient 2)
Duration (min)	210	360 (patient 1 and patient 2)	168	104–114 (patient 1) 116–177 (patient 2)
Blood flow (mL/min)	300	300 (patient 1 and patient 2)	30–40	40–50 (patient 1) 40–60 (patient 2)
Dialysate/Plasma flow (mL/min)	360	500 (patient 1 and patient 2)	20–30	25–40 (patient 1 and patient 2)
Dialysate Ca^2*^ (mM)	Patient 1: 1.25	Patient 1: 1.25 Patient 2: 1.0–1.25	–	–

#### Follow-up and outcomes

During follow-up, there was a subsequent increase in plasma 25-OH-vitamin D_3_ and calcium concentrations in the patient. Upon relapse of hypercalcemia at 3.2 mmol/L, she was again hospitalized and underwent forced diuresis with torasemide and administration of another dose of ibandronic acid (2 mg) intravenously. This corrected the hypercalcemia and the patient was discharged on loop diuretics. Two months later, there was another relapse of hypercalcemia (3.1 mmol/L) in the setting of persistence of elevated plasma concentrations of 25-OH-vitamin D_3_ (476 nmol/L). 24-h urine collection revealed increased calcium excretion which prompted us to reduce the patient's calcium intake through a low calcium diet. Using tap water with a commercial liquid filter system (Brita^®^ MAXTRA +), the calcium concentration decreased by 90% from 1.94 mmol/L to 0.2 mmol/L. After 9 months of follow-up, kidney function stabilized at an eGFR-CKD-EPI of 36 mL/min/1.73m^2^ (plasma creatinine level of 141 µmol/L or 1.6 mg/dL), while the plasma calcium concentration and the vitamin D_3_ metabolites were (almost) normalized (Fig. 1).

### Case 2

#### Patient information

A 33-year-old male initially visited his family doctor because of persistent nausea, vomiting, headache, stomach pain and drowsiness, as well as polyuria and polydipsia. Laboratory examination performed in a different hospital showed life-threatening hypercalcemia, leading to emergency admission of the patient. He had taken up to 35,000 I.U./days of vitamin D3 over the previous 6–12 months (total approximately 10,000,000 I.U.) to enhance the expected beneficial effect related to vitamin D reported in the media.

#### Clinical findings

Elevated blood pressure up to 200 mmHg systolic was detected, while in all other organ systems there were no abnormal findings that could be attributed to hypercalcemia.

#### Diagnostic assessment

**Fig. 2:**
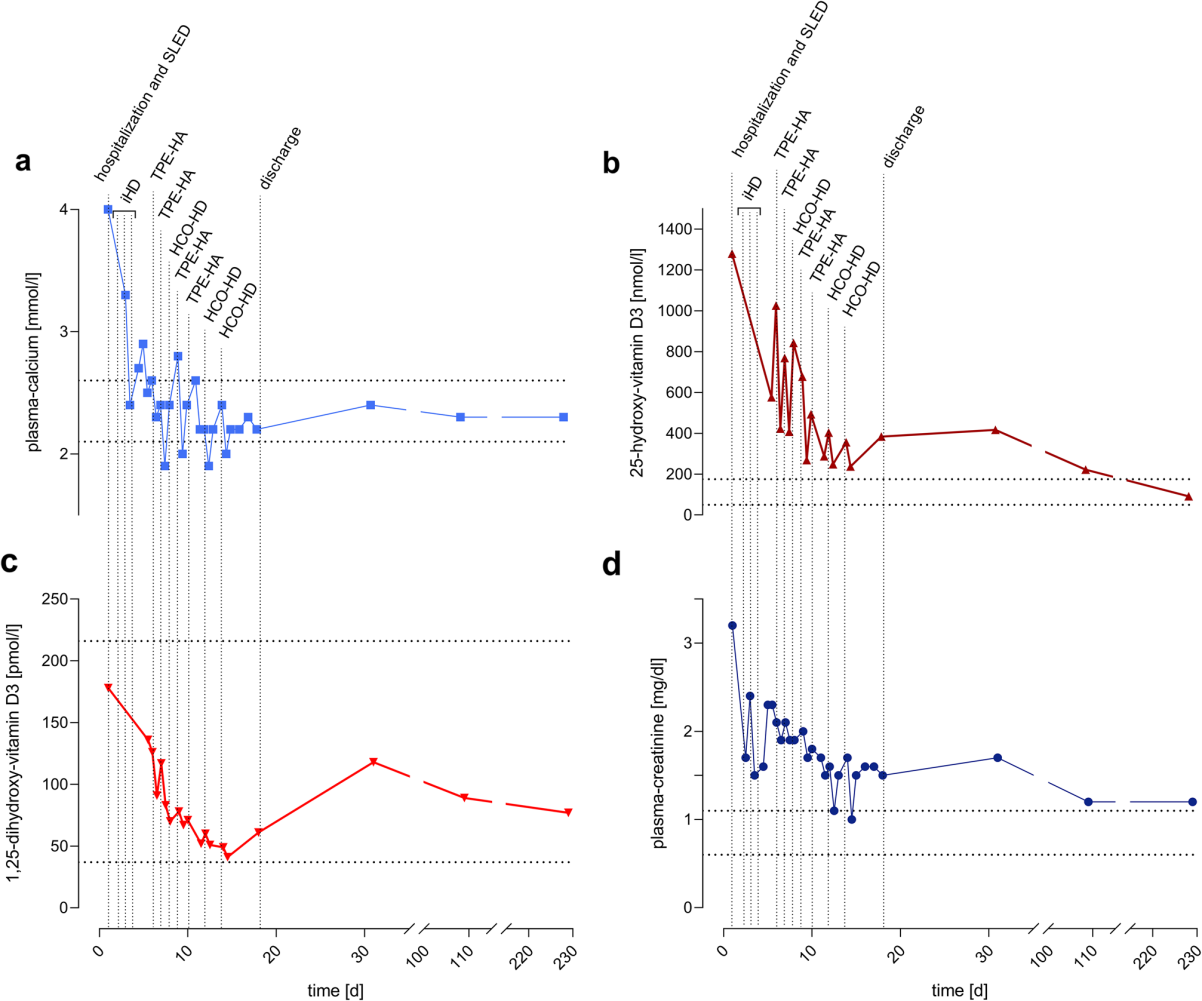
Time course of plasma concentrations of calcium (**a**), 25-OH-vitamin D_3_ (**b**), 1,25-OH-vitamin D_3_ (**c**) and creatinine (**d**) of patient 2. Hospital admission, discharge and extracorporeal treatments are marked with vertical dotted lines. Normal ranges are marked with horizontal dotted lines. *HCO–HD* high cut-off hemodialysis, *TPE-HA* total plasma exchange with replacement by human albumin, *SLED* sustained low efficiency dialysis, *iHD* intermittent hemodialysis

**Fig. 3:**
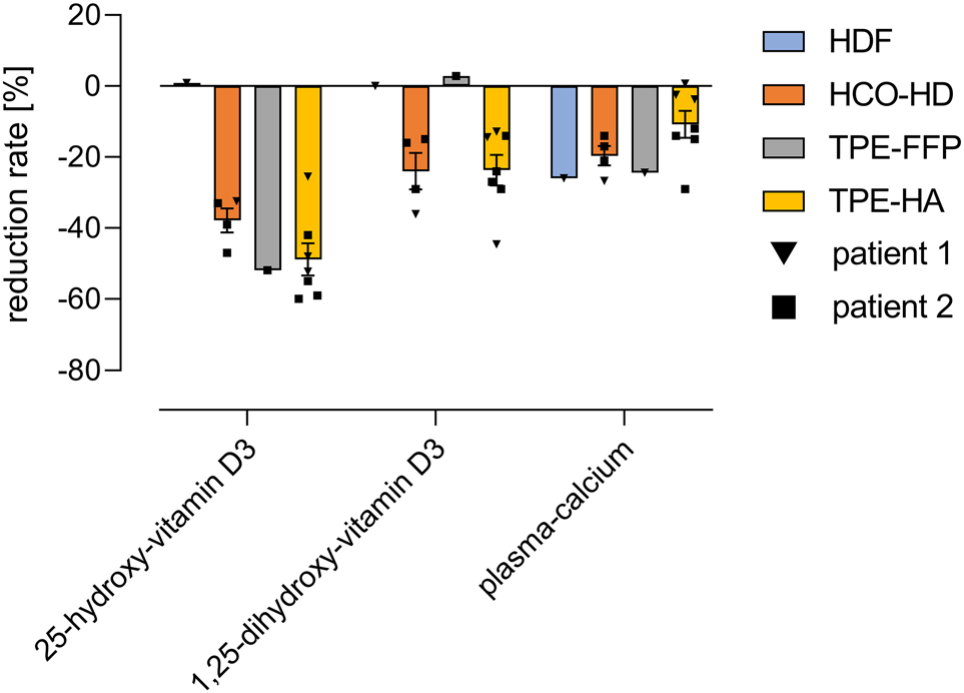
Reduction rates of plasma 25-OH-vitamin D_3_, 1,25-OH-vitamin D_3_ and calcium concentrations achieved with the different extracorporeal treatments of patient 1 and patient 2. Arithmetic means with standard error of the mean (SEM). *HDF* hemodiafiltration, *HCO–HD* high cut-off hemodialysis, *TPE-FFP* total plasma exchange with replacement by fresh frozen plasma, *TPE-HA* total plasma exchange with replacement by human albumin

Hypercalcemia with total and ionized plasma calcium concentrations of 4.0 mmol/L and 2.19 mmol/L (Fig. 2A),respectively, as well as an elevated plasma concentration of 25-OH-vitamin D3 of 1289 nmol/L (Fig. 2B) and normal concentrations of 1,25-OH-vitamin D3 of 178 pmol/L (Fig. 2C) were detected. An increased plasma creatinine concentration of 193 µmol/L or 2.2 mg/dL (reference < 97 µmol/L or < 1.1 mg/dL) indicated acute kidney injury (Fig. 2D). There was no evidence of a pre-renal or post-renal condition, malignancy or primary hyperparathyroidism. Figure 3 describes the removal of 25-vitamin-D, 1,25-vitamin D and calcium performing different methods like hemodialysis for example.

#### Therapeutic intervention

The patient was transferred to the intensive care unit due to the life-threatening situation and, after inserting a central venous catheter, was immediately treated with sustained low efficiency dialysis (one session) and intermittent hemodialysis (three sessions) to correct hypercalcemia (Fig. 2A). In addition, torasemide (5–20 mg/days), intravenous fluids (3.0–6.7 L/days) and 3 mg ibandronic acid were administered. Based on our prior experience with patient 1, we focused treatment on total plasma exchange with humanalbuimin and high cut-off hemodialysis to eliminate vitamin D_3_ metabolites. As described above, we performed four sessions of therapeutic plasma exchange with human albumin with an exchange volume of 2.5–3.5 L corresponding to 0.61–0.85 times the plasma volume, and three 6 h sessions of high cut-off hemodialysis. Treatment with therapeutic plasma exchange with human albumin resulted in a decrease of 25-OH-vitamin D_3_ and 1,25-OH-vitamin D_3_ by 42–60% and 14–29%, respectively. On alternate days, we performed high cut-off hemodialysis achieving reduction rates of 33–47% for 25-OH-vitamin D_3_ and 15–29% for 1,25-OH-vitamin-D_3_. To investigate the efficacy of both treatments, we analyzed the plasma eluate of plasma exchange with human albumin and spent dialysate from high cut-off hemodialysis. We found that, depending on the initial value, the amount removed was between 1046 and 3168 nmol 25-OH-vitamin D_3_ for plasma exchange with human albumin (four measurements) and 660 nmol (one measurement) for high cut-off hemodialysis. Regarding 1,25-OH-vitamin D_3_, total plasma exchange with human albumin removed 162–510 pmol, and high cut-off hemodialysis removed 134 pmol. The total removed amount of calcium in high cut-off hemodialysis was 36 mmol and up to 6-times higher compared to total plasma exchange with human albumin (max. 6–9 mmol). The patient was discharged with normal plasma calcium and 1,25-OH-vitamin-D_3_ concentrations, while plasma 25-OH-vitamin-D_3_ concentration was still elevated (383 nmol/L; Fig. 2B).

#### Follow-up and outcomes

The patient recovered normal kidney function after 109 days while maintaining normocalcemia (Fig. 2A, D). The 25-OH-vitamin D_3_ and 1,25-OH-vitamin D_3_ levels normalized at 91 nmol/L and 77 pmol/L, respectively, after 229 days (Fig. 2B, C).

## Discussion

These two cases suggest that removal of excessive amounts of vitamin D_3_ metabolites is feasible using extracorporeal treatments. The most effective treatment modalities were total plasma exchange with human albumin and high cut-off hemodialysis (Table 1). Compared to high cut-off hemodialysis, a single session plasma exchange with human albumin was more effective in removing 25- and 1,25-OH-vitamin D_3,_ while on the other hand high cut-off hemodialysis removed more calcium. This is in contrast to high-flux hemodiafiltration, which reduced plasma calcium concentrations effectively, but had no effect on either vitamin D_3_ metabolites. This was not surprising, as vitamin D_3_ bound to VDBP is too large to pass through the high-flux hemodialysis membrane [1].

Due to the lipophilicity of vitamin D_3_ metabolites, a large proportion is stored outside the bloodstream, e.g., in the adipose tissue [3], which explains the rebound after the end of the extracorporeal treatments and the long time needed for normalization of plasma 25-OH-vitamin D_3_ concentrations. Still, extracorporeal treatments rapidly reduce excessive concentrations of 25-OH-vitamin D_3,_ which have been shown to directly activate the vitamin D receptor, thereby maintaining hypercalcemia [7]. The main drawbacks of extracorporeal treatments are the risks related to the procedures, longer hospitalization and increased costs.

In summary, these cases suggest that extracorporeal treatments such as high cut-off hemodialysis and total plasma exchange with human albumin may be considered as add-on treatments in refractory cases of vitamin D_3_-induced hypercalcemia and excessive concentrations of 25-OH-vitamin D_3_, since they do not only treat hypercalcemia, but also causative hypervitaminosis. Further research is needed to define the optimal indication, dose and timing of extracorporeal treatments in vitamin D_3_-induced hypercalcemia.
